# Cell Edges Accumulate Gamma Tubulin Complex Components and Nucleate Microtubules following Cytokinesis in *Arabidopsis thaliana*

**DOI:** 10.1371/journal.pone.0027423

**Published:** 2011-11-09

**Authors:** Chris Ambrose, Geoffrey O. Wasteneys

**Affiliations:** Department of Botany, The University of British Columbia, Vancouver, British Columbia, Canada; Umeå Plant Science Centre, Sweden

## Abstract

Microtubules emanate from distinct organizing centers in fungal and animal cells. In plant cells, by contrast, microtubules initiate from dispersed sites in the cell cortex, where they then self-organize into parallel arrays. Previous ultrastructural evidence suggested that cell edges participate in microtubule nucleation but so far there has been no direct evidence for this. Here we use live imaging to show that components of the gamma tubulin nucleation complex (GCP2 and GCP3) localize at distinct sites along the outer periclinal edge of newly formed crosswalls, and that microtubules grow predominantly away from these edges. These data confirm a role for cell edges in microtubule nucleation, and suggest that an asymmetric distribution of microtubule nucleation factors contributes to cortical microtubule organization in plants, in a manner more similar to other kingdoms than previously thought.

## Introduction

Eukaryotic cell complexity depends on diverse and complex three-dimensional microtubule (MT) configurations, which play key roles in cell division and the establishment of cellular polarity. Both animal and plant cells use centralized MT nucleators to establish radial MT arrays; in animals this is the centrosome, and in plants the nuclear envelope [1]. Although centrosomal-based nucleation dominates many stages of animal cell growth and development, in plant cells, nuclear MT initiation appears to be restricted largely to pre- and post-mitotic MT arrays [2]. Later, during cell expansion and after growth cessation, MT nucleation occurs predominantly in the cell cortex. Evidence for this includes the ability of cortical microtubule arrays to recover following drug-induced disassembly during late stages of interphase, when there is no apparent contribution from microtubules initiated at the nuclear complex [3], [4], [5], [6] or the assembly of MTs from purified brain tubulin at cortical sites in permeabilized cells [7]. Initiation of MTs in the cell cortex can occur at dispersed sites [8], [9], [10] or from pre-existing MTs, either diverging at an angle [4], [11], [12] or running parallel to [13], [14] the pre-existing microtubules.

In 1978, Gunning et al described complexes of microtubules in vesicle-rich regions along edges of root apical cells in the water fern *Azolla* [15]. Based on these observations and the fact that the putative nucleating sites were conspicuous only when cortical arrays, including preprophase bands, were being established [16], it was hypothesized that cortical microtubules are nucleated at cell edges and that these nucleating zones may contribute to the parallel orientation of cortical MTs on different faces of polyhedral plant cells [15], [16]. Later studies also found dense accumulations of vesicular elements specifically at the outer cell edge of newly formed cell walls, and these were intersected by large MT bundles [17], [18]. To date, however, no known MT nucleating factors have been observed at cell edges.

We recently showed that newly divided cells of root meristematic zones and unexpanded leaves in *Arabidopsis thaliana* contain large MT bundles, which intersect the sharp edges adjoining the newly formed cell wall and the outer periclinal wall [19]. Cortical MTs encountering these sharp edges undergo frequent catastrophe unless the MT-associated protein CLASP is present. When situated at specific cell edges, CLASP counteracts MT catastrophe induction and promotes the establishment of the large MT bundles spanning adjacent cell faces. In these cells, MT growth is predominantly toward these edges, although, in keeping with the transfacial nature of the MT bundles, some incidence of MT growth away from edges was also observed [19].

Here we set out to determine if the prominent transfacial MT bundles found at newly formed cell edges in Arabidopsis contain known components of MT nucleating complexes, and whether they serve to initiate MTs on cell faces. We confirm that post-cytokinetic cell edges in leaves and roots of *Arabidopsis thaliana* contain enrichments of gamma-tubulin complex components GCP2 and GCP3. MT nucleation from these post-cytokinetic cell edges was observed in meristematic root cells and was especially prominent in young leaf epidermal cells.

## Results

### Gamma Tubulin Complex Proteins localize to newly formed cell edges

Based on our previous observations of MT growth away from the edges that form between the new cross wall and the outer periclinal face of epidermal cells [19], we hypothesized that newly formed edges are enriched with MT nucleation complex components. The conserved gamma tubulin complex proteins 2 and 3 (GCP2 and GCP3, respectively) are confirmed to be present in plants [20] and have been shown to help coordinate the assembly of cortical microtubule arrays [21]. In this study, we examined the distribution of gamma tubulin complexes in root tip meristematic zones and in unexpanded leaf epidermal cells using transgenic lines expressing green fluorescent protein-tagged versions of GCP2 and 3 [21]. Data are shown for GCP2-GFP, although identical distribution patterns were observed with the GCP3-GFP reporter (Fig. S1), supporting the fact that they are part of the same functional complex.

**Figure 1:**
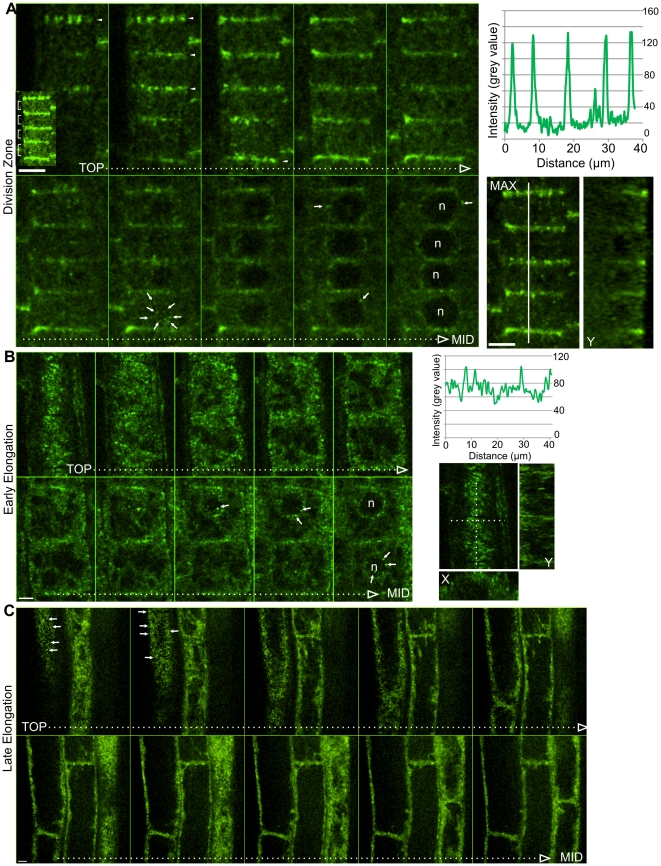
Localization of GCP2-GFP in roots. **A** Post-cytokinetic epidermal root division zone cells. Sequential images from confocal stack, starting at outer periclinal face, ending at median optical plane. Right panel shows a maximum Z projection of the series, a Y-axis orthogonal view, and a fluorescence intensity plot corresponding to the white line. **B** Root epidermal cells from early elongation zone. Sequential images from confocal stack, starting at outer periclinal face, ending at median optical plane. Right panel shows the outermost optical slice of the series, X- and Y-axis orthogonal views, and a fluorescence intensity plot corresponding to the dotted line. **C** Root epidermal cells from the late elongation zone. Sequential images from confocal stack, starting at outer periclinal face, ending at median optical plane. Arrowheads indicate edge enrichment. Arrows indicate perinuclear and cortical punctae. n = nucleus. Confocal planes correspond to 0.5 µm slice intervals. Scale Bars = 5 µm.

In root tips, strong enrichment of GCP2-GFP was found at the sharp edge separating the newly-formed cross wall from the outer periclinal surface (Fig. 1A). GCP2 enrichment was restricted to the outer periclinal edge. The other edges shared between the newly formed wall and parental walls lacked any noticeable enrichment). Edge enrichment was non-uniform along edges; appearing as distinct punctae of various shapes and sizes, which is reminiscent of the CLASP and transfacial bundle patterns previously described [19]. Edge enrichment is most clearly illustrated in the sequential frames from a confocal Z-stack shown in figure 1A. Slicing deeper into the cells shows a decreasing fluorescence signal intensity along the transverse cross walls but relatively constant fluorescence elsewhere in the cells (Orthogonal view in Fig. 1A), which demonstrates the strong enrichment of GCP2 at the transverse edge at the outer periclinal face.

**Figure 2:**
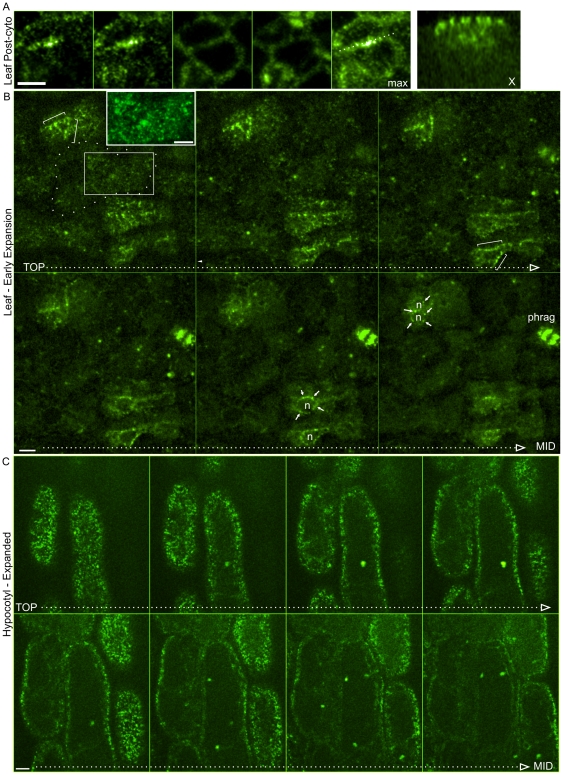
Localization of GCP2-GFP in cotyledons and hypocotyls. **A** Cotyledon post-cytokinetic epidermal cells. Sequential images from confocal stack, starting at outer periclinal face, ending near the inner periclinal face. Right panel shows a maximum Z projection of the series and X-axis orthogonal view corresponding to the dotted line. Brackets indicate edge enrichment in post-cytokinetic cells. Arrows indicate perinuclear punctae. **B** Post cytokinetic and early expanding cotyledon epidermal cells. Cells at multiple stages are present. Sequential images from confocal stack, starting at outer periclinal face, ending near the median optical plane. Inset in B shows high contrast image corresponding to boxed region. n = nucleus. Phrag = phragmoplast. **C** Expanded hypocotyl epidermal cells. Sequential images from confocal stack, starting at outer periclinal face, ending near the median optical plane. Confocal planes correspond to 1 µm slice intervals. Scale Bars = 5 µm.

In addition to edge distribution, we observed GCP2-GFP as perinuclear and cortical punctae (Fig. 1B and C; Fig. S2). The temporal progression of these three distinct localizations is as follows (illustrated in Fig. S2). In root tip division zone cells, all three localizations were present at the same time within each single cell. The perinuclear enrichment appeared first, becoming apparent late in cytokinesis, before phragmoplast fusion with the parental cortex. Following full dissolution of the phragmoplast, edge accumulation and cortex localization appeared after a short period during which only perinuclear accumulation was seen (indicating that edge accumulation is not due to phragmoplast remnants). Edge accumulation persisted in all division zone cells. As cells entered the elongation zone, edge enrichment was lost, while nuclear and cortical localizations persisted. Finally, during mid-phase elongation, nuclear localization was lost, and only the cortical punctae remained (Fig. 1B and C). In cotyledons and leaves, as in roots, the GCP2 edge enrichment was restricted to early post-cytokinetic cells (Fig. 2A). The perinuclear signal was more short-lived than in roots, disappearing soon after division, while the edge signal remained until cell expansion onset (Fig. 2B). As previously shown, we observed punctate cortical fluorescence in leaf cells. We observed similar cortical distribution along the outer periclinal faces of expanded hypocotyl cells (Fig. 2C), as previously reported [21].

### Assessment of MT growth polarities with respect to post-cytokinetic edges

**Figure 3:**
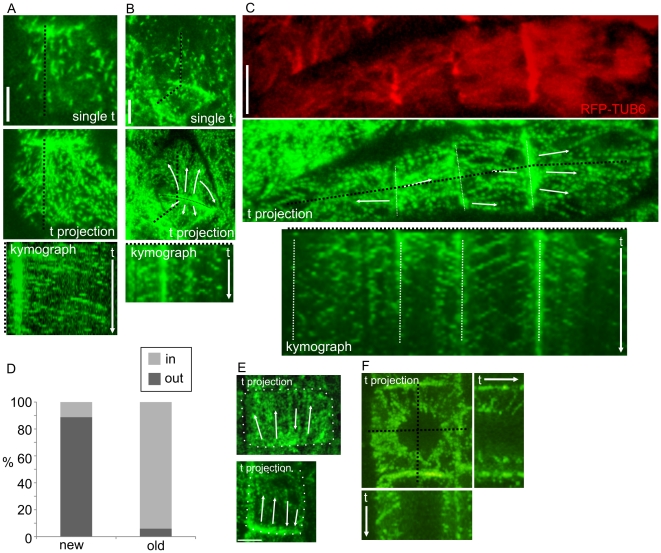
MT growth directions in post-cytokinetic cells. (A–C) EB1b-GFP in post-cytokinetic cotyledon epidermal cells. For A and B, the top images are single time points, the middle panels are time projections of the same cells, and the bottom panels are kymographs corresponding to the dotted black lines in the top and middle panels. **A** Recently divided pavement epidermal cell. **B** A second example of recently divided cells. MTs are difficult to visualize due to high autofluorescence from vacuolar anthocyanins present in young cells. These cells are from the guard cell lineage. **C** Top (red) panel shows time projection of RFP-TUB6 corresponding to the time projection of EB1b-GFP in the middle panel. **D** Quantification of growth polarities with respect to newly formed and old cell edges in cotyledon pavement epidermal cells. **E** Post-cytokinetic root epidermal division zone cells. Two examples are shown, both show nucleation primarily from the bottom edge, and both contain multiple polarities with respect to these edges. Dotted lines indicate cell outlines. **F** Time projection and corresponding X and Y kymographs of EB1b-GFP showing nuclear initiation. Projections are taken from XYTZ series with 5 second intervals, and are 5 µm thick projections. Scale Bars = 5 µm.

We used the microtubule plus end tracker EB1b-GFP to assess MT growth polarities with respect to post-cytokinetic edges in root division zone cells and unexpanded post-cytokinetic leaf epidermal cells. In agreement with enrichment of nucleating factors at these edges, we observed abundant EB1b-GFP spots emerging from and moving away from the newly formed edges (Fig. 3). This behaviour was particularly striking in leaf epidermal cells, and is well illustrated using time projections (which result in dotted lines corresponding to EB1 trajectories), and kymographs, which show slanted lines corresponding to movement of EB1b-GFP tracks along the line from which the kymograph was generated (Fig. 3A–C).

Typically, EB1b-GFP spots emerged from distinct regions along the post-cytokinetic edges (Fig. 3A–C; Movie S1), in agreement with the non-uniform distribution of GCP2. Coexpression of EB1b-GFP with Ubiquitin1 promoter driven RFP-TUB6 in young cotyledons showed that these emergence sites corresponded to points where MT bundles intersected the edges (Fig. 3C). These polarized emergence sites dominated most of the newly formed edge. Within these same edges, MT bundles were sometimes found that contained MTs growing predominantly toward the edges, or in mixed orientations, although this was relatively infrequent, and typically occurred at the outermost regions of the new edge (see arrows in Fig. 3C). Quantification of EB1b-GFP trajectories within the polarized emergence sites with respect to newly formed edges showed that 88.8±2.4% of EB1b-GFP spots moved away from these edges, while 11.2±2.4% moved toward them (Fig. 3D; n = 12 cells; 250 MTs). This dominant polarity of trajectories away from newly formed edge extended across the cell and reached the opposite (older) cell edge, where 94.1±1.4% of EB1b-GFP spots moved into them, while 5.9±1.4% moved out (Fig. 3D: n = 12 cells, 221 MTs). At the onset of cell expansion, edge initiation was lost, and EB1b-GFP tracked predominately toward, parallel to, or in mixed orientations relative to new edges (Fig. S3).

In roots, we also observed abundant movement of EB1 spots away from new edges (Fig. 3E), although observation was relatively difficult because most division zone epidermal cells were occluded by the overlying lateral root cap cells, which are elongated and exhibit high expression of 35s:EB1b-GFP relative to the inner tissues. We also observed emergence of EB1 spots at the nuclear surface followed by trajectories toward the cell cortex, indicating MT plus end polymerization in that direction (Fig. 3F; Movie S2). This is in agreement with the localization of GCP2-GFP on the nuclear surface within division zone and early elongation zone cells. In these cells, nuclear initiation prevailed over edge nucleation, which may contribute to the relatively infrequent observation of edge nucleation in roots when compared to leaves (which exhibit a shorter period of GCP perinuclear distribution).

## Discussion

The ability of cell edges to concentrate MT nucleating activity at a discrete cellular locale to effect cell-wide MT organization and polarity is consistent with the properties of a MT organizing center (MTOC). We show that GCP accumulates along newly formed cell edges at sites intersected by transfacial MT bundles, and that EB1 spots within these bundles follow trajectories predominantly away from the new edge. During this stage, GCP edge accumulation is accompanied by perinuclear and cortex localization. Edge enrichment disappears when cells enter the elongation zone, while perinuclear accumulation persists until mid-phase expansion. We recently showed that the MT-associated protein CLASP is present at cell edges [19]. The difference is that CLASP remains on edges longer than GCP, persisting often until midway through the elongation zones. These patterns match the observed MT growth polarities with respect to newly formed edges: when GCP and CLASP are present, outward growth dominates; when GCP is lost and CLASP remains, mixed/inward growth dominates. Later, when CLASP is no longer at these edges, MTs are unable to bypass these sharp edges, which results in transverse MT arrays. Loss of perinuclear initiation during the elongation phase also presumably favours transverse MT orientation, since nuclear initiated endoplasmic MTs have been shown to play a role in array randomization [22].

In Gunning et al's original hypothesis, it was suggested that MT nucleating capacity could be conferred even prior to mitosis by the preprophase band [15], which lies along the future cell edges. This remains an obvious possibility but unlike the MT distribution patterns described in Azolla roots [15], GCP distribution patterns observed in the current study were restricted to the outer edges and therefore, a pattern overlapping only partly with the prior preprophase band.

What is the function of nucleation at young post-cytokinetic cell edges? We recently showed that transfacial MT bundles contribute to maintaining the sharp curvature of these edges [19]. Loss of these bundles in the *clasp-1* mutant is associated with premature bowing of these cells, presumably due to insufficient fortification of the cell walls adjoining the new edge [19]. Edge nucleation may assist in establishment and/or maintenance of transfacial bundles, which then contribute to localized cell wall fortification. In general, plants deposit wall materials in a more or less uniform fashion throughout the cell cortex. There are, however, many instances in which localized wall thickening is required, such as at guard cell pores, the indentations in leaf pavement cells, transfer cells and tracheary elements. Our data here suggest that localized wall thickening is also important at newly-formed walls. Dense vesicular matter and gamma tubulin accumulate at guard cell pore sites, and are intersected by dense MT bundles, particularly just after cytokinesis, and match the orientation of cellulose microfibrils [17], [18], [23], [24]. The thickenings at indentation sites along the sides of leaf epidermal cells also contain vesicular matter and dense MT bundles, which are required for their formation [25], [26]. Depletion or removal of gamma tubulin results in failures in both guard cell pore formation as well as pavement cell lobing [27]. It is therefore logical that localized wall thickening should also occur at the newly formed post-cytokinetic walls discussed in the current study. The observations of dense MT bundles [17], [18], [19] and the accumulation of MT initiation components shown here support this hypothesis.

## Methods

### Plant material and growth conditions

*Arabidopsis thaliana* Columbia ecotype plants were grown in continuous light conditions on vertical agar plates containing Hoagland's medium. For cotyledon cells, we used 4–5 day old seedlings. For root cells, we used 5–7 day seedlings. In both cases, plants were mounted on coverslips and covered with a 1–2 mm slice of 1% bacto-agar inside Petri dish chambers.

### Microscopy and image analysis

Images were acquired on a Perkin-Elmer spinning disk microscope. Images were processed using imageJ software (http://rsb.info.nih.gov/ij/), and figures were assembled using Corel Draw software. pGCP2:3XGFP and pGCP3:GCP3-GFP expressing plants were obtained from Prof. Takashi Hashimoto [21]. EB1b-GFP was driven under the 35S promoter to allow for visualization in root tips and unexpanded leaves, since the native EB1b promoter does not express in these cells in our experience. Expression of RFP-TUBULIN6 (RFP-TUB6) was driven by the Ubiquitin 1 promoter [19]. For quantification of EB1b-GFP growth trajectories relative to newly formed cell edges, only EB1 spots that emerged from, or tracked completely into, were used.

## Supporting Information

Figure S1 Localization of GCP3-GFP to cell edges Shown is a confocal Z series from the outer cotyledon surface into the epidermal cell midplanes. Arrowheads indicated enrichment at new cell edges. Dotted line indicates direction of sectioning. Confocal planes correspond to 0.5 µm slice intervals. Scale Bars = 5 µm.
(TIF)

Figure S2 Localization patterns of GFP-GCP2 in epidermal root division zone cells Sequential images from confocal stack, starting at outer periclinal face, ending at inner optical plane. Right panel shows a maximum Z projection of the series, and a Y-axis orthogonal view (corresponding to dotted line). Four cells are shown. The top two have just completed cytokinesis, and contain perinuclear accumulation and cortical localization (arrows indicate several punctae), but lack edge accumulation (new edge is indicated by arrowheads). Middle cell contains mitotic spindle in metaphase. Note lack of cortical signal. Bottom cell is telophase/late cytokinesis just prior to cell plate fusion. Phragmoplast is still present and perinuclear accumulation has appeared, while cortical localization and edge enrichment are not yet present. n = nucleus. Confocal planes correspond to 0.5 µm slice intervals. Scale Bars = 5 µm.
(TIF)

Figure S3 EB1b-GFP tracking directions relative to newly formed edges in cells entering the elongation zone **A** Cells with EB1 tracking predominately parallel to newly formed edge. Single timepoint and time projection shown. **B** Cells with mixed EB1 directions relative to new cell edge. Single timepoint and time projection shown. Dotted lines indicate new cell edges. Arrows indicate EB1b-GFP spot direction. Confocal planes correspond to 0.5 µm slice intervals. Scale Bars = 5 µm.
(TIF)

Movie S1 EB1b-GFP spots emerge from newly formed cell edges Leaf epidermal cells corresponding to Figure 3b. Left panel is EB1b-GFP, right panel is RFP-TUB6 and middle panel is merged image with EB1 colored red, and RFP-TUB6 colored green. Time interval is 12 seconds between frames. Movie plays at 12 fps.
(MOV)

Movie S2 EB1b-GFP spots grow away from the nucleus Focal midplane of a root epidermal cell from the late division zone, corresponding to figure 3F. Left panel is normal time-lapse, and right panel is a running average (3 frame averaging) of the same cell. Running average displays assist observation of the directionality of EB1 spot movement. Time intervals between frames is 5 seconds. Movie plays at 12 fps.
(MOV)
