# Supplementary figures and images for: Cell Edges Accumulate Gamma Tubulin Complex Components and Nucleate Microtubules following Cytokinesis in Arabidopsis thaliana
Source: PLoS One. 2011 Nov 9;6(11):e27423. doi: 10.1371/journal.pone.0027423 (PMC3212562; doi:10.1371/journal.pone.0027423)

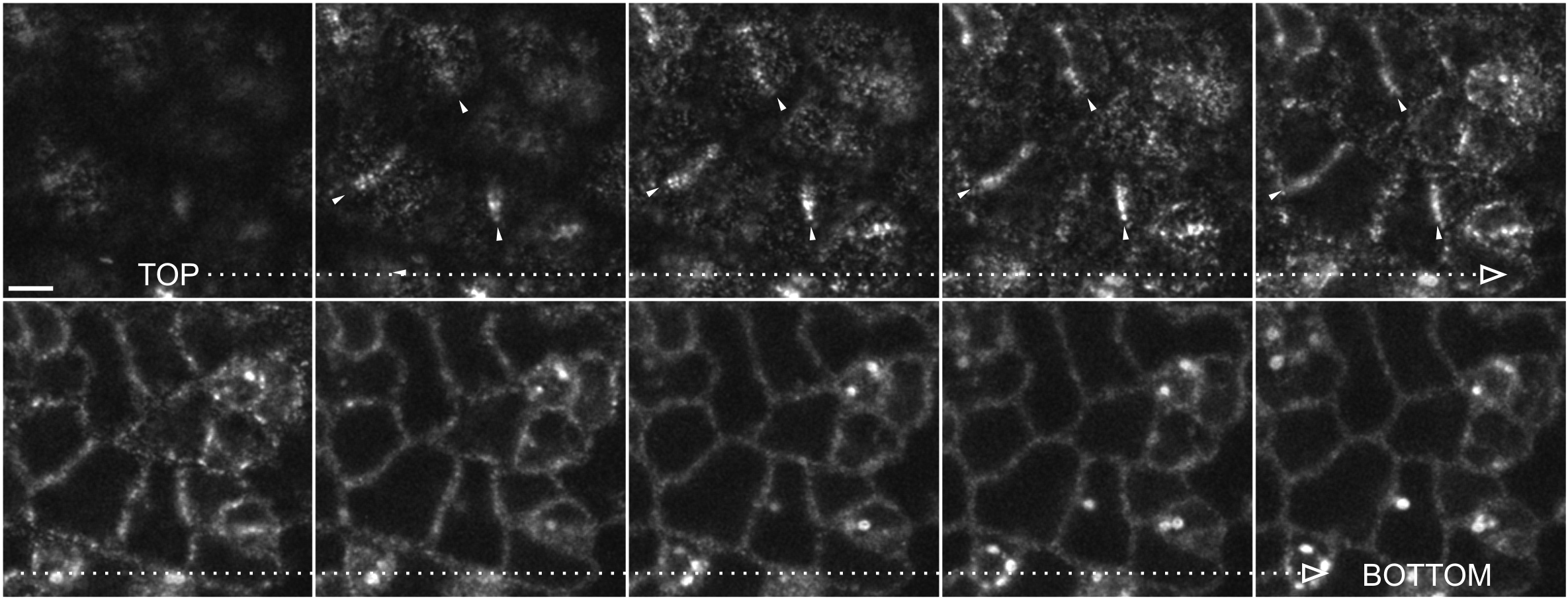

Supplement: Figure S1 — Localization of GCP3-GFP to cell edges Shown is a confocal Z series from the outer cotyledon surface into the epidermal cell midplanes. Arrowheads indicated enrichment at new cell edges. Dotted line indicates direction of sectioning. Confocal planes correspond to 0.5 µm slice intervals. Scale Bars = 5 µm. (TIF) [file pone.0027423.s001.tif]

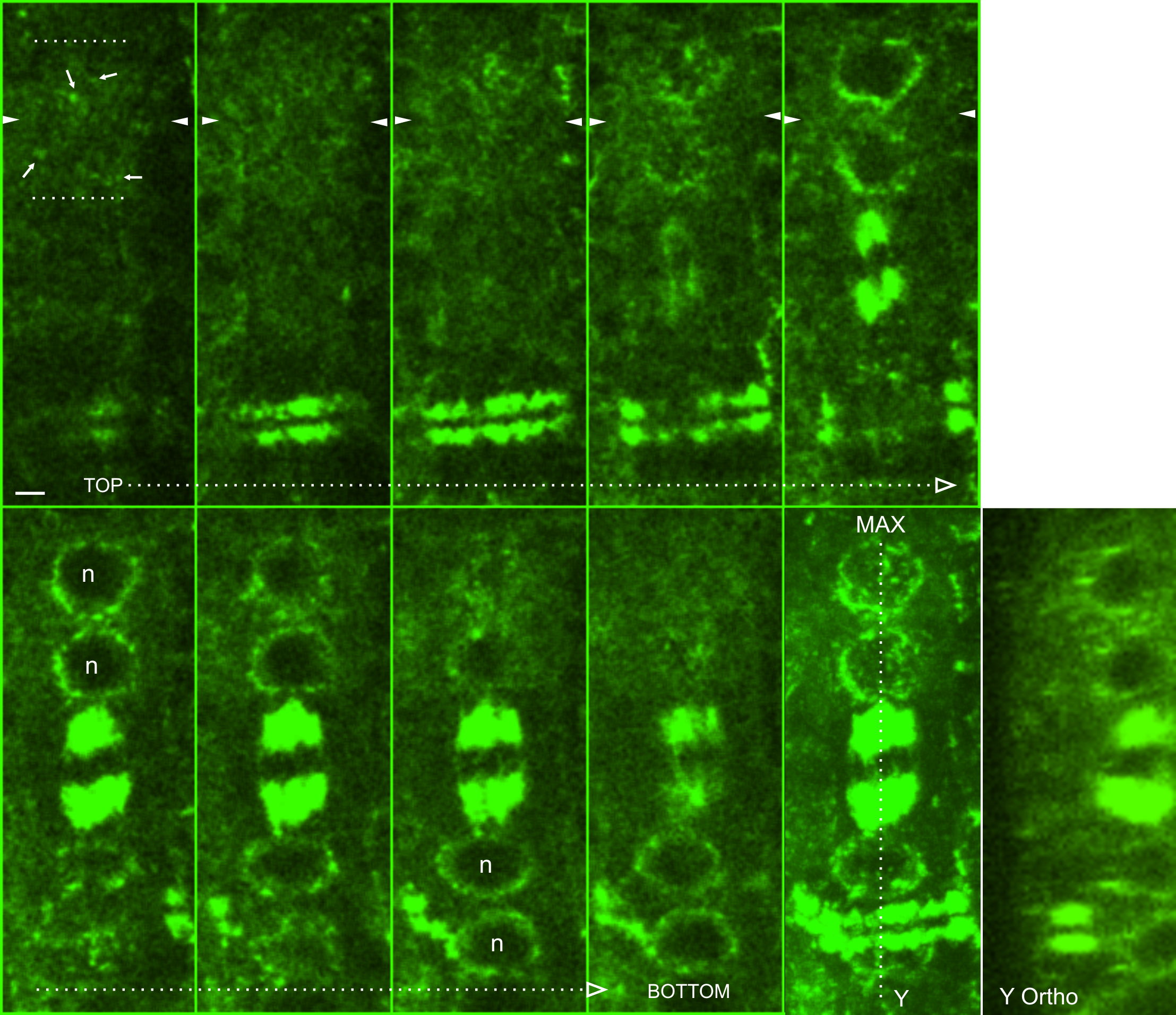

Supplement: Figure S2 — Localization patterns of GFP-GCP2 in epidermal root division zone cells Sequential images from confocal stack, starting at outer periclinal face, ending at inner optical plane. Right panel shows a maximum Z projection of the series, and a Y-axis orthogonal view (corresponding to dotted line). Four cells are shown. The top two have just completed cytokinesis, and contain perinuclear accumulation and cortical localization (arrows indicate several punctae), but lack edge accumulation (new edge is indicated by arrowheads). Middle cell contains mitotic spindle in metaphase. Note lack of cortical signal. Bottom cell is telophase/late cytokinesis just prior to cell plate fusion. Phragmoplast is still present and perinuclear accumulation has appeared, while cortical localization and edge enrichment are not yet present. n = nucleus. Confocal planes correspond to 0.5 µm slice intervals. Scale Bars = 5 µm. (TIF) [file pone.0027423.s002.tif]

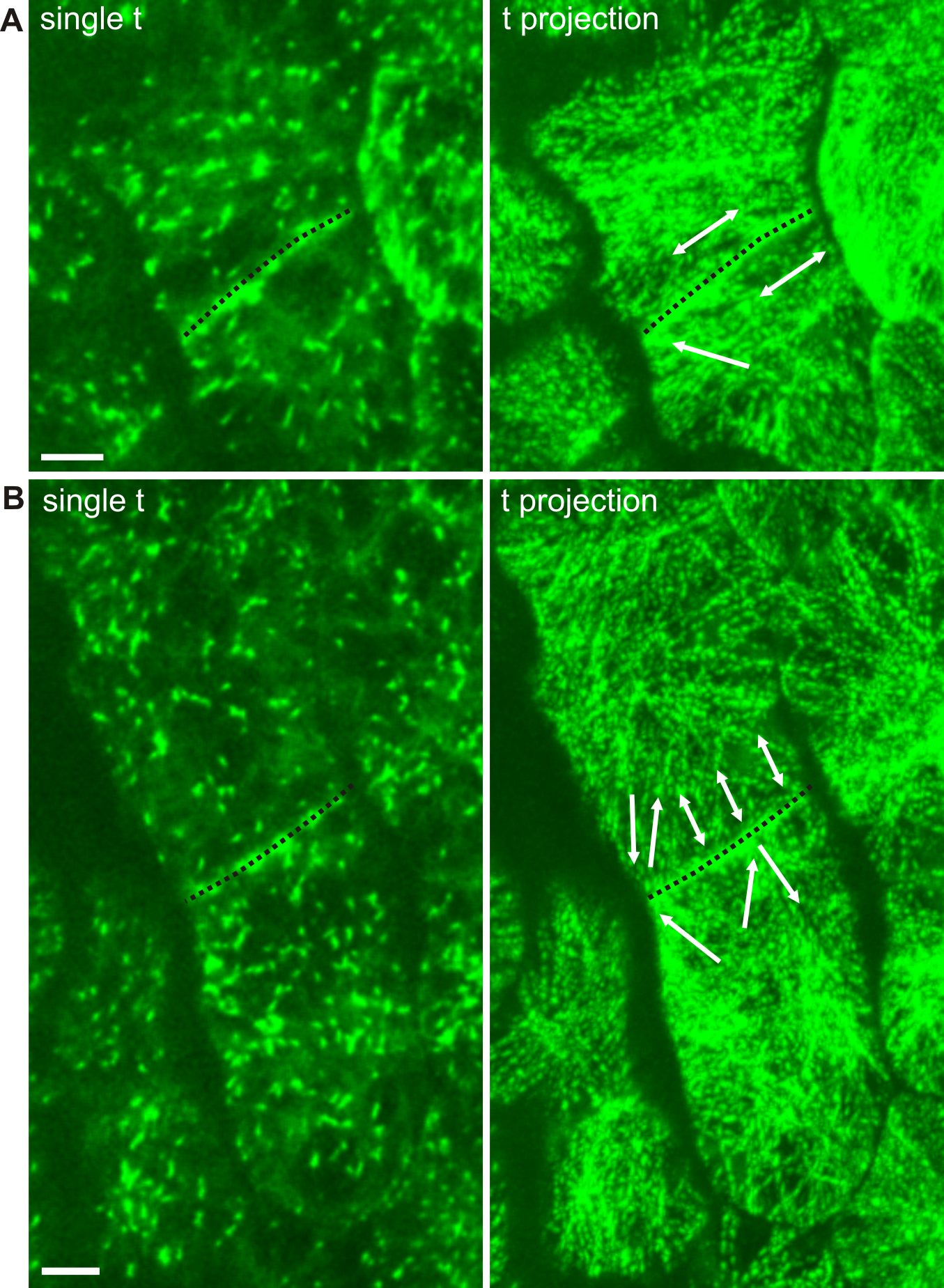

Supplement: Figure S3 — EB1b-GFP tracking directions relative to newly formed edges in cells entering the elongation zone A Cells with EB1 tracking predominately parallel to newly formed edge. Single timepoint and time projection shown. B Cells with mixed EB1 directions relative to new cell edge. Single timepoint and time projection shown. Dotted lines indicate new cell edges. Arrows indicate EB1b-GFP spot direction. Confocal planes correspond to 0.5 µm slice intervals. Scale Bars = 5 µm. (TIF) [file pone.0027423.s003.tif]
